# Methanolic neem (*Azadirachta indica*) stem bark extract induces cell cycle arrest, apoptosis and inhibits the migration of cervical cancer cells in vitro

**DOI:** 10.1186/s12906-022-03718-7

**Published:** 2022-09-10

**Authors:** Saurav Kumar, Vaishali Mulchandani, Jayasri Das Sarma

**Affiliations:** Department of Biological Sciences, Indian Institute of Science Education and Research Kolkata, Mohanpur, 741246 India

**Keywords:** Cervical cancer, Methanolic Neem Bark Extract, Cell cycle arrest, Apoptosis, Metastasis

## Abstract

**Background:**

Cervical cancer remains one of the significant causes of mortality in women due to the limitations of current treatment strategies and their associated side effects. Investigation of alternative medicine, including phytomedicine, has shown effective anti-cancer potential with fewer side effects. *Azadirachta indica* (commonly known as neem) is known for its medicinal properties. The present study investigated the anti-cancer potential of methanolic neem stem bark extract (MNBE) against cervical cancer using HeLa, SiHa, and ME-180 cell lines.

**Methods:**

Cytotoxic effect of MNBE on cultured cell lines was evaluated by MTT and clonogenic assay. The growth-inhibiting effect of MNBE was further confirmed by performing cell cycle analysis and apoptosis assay using flow cytometry. The anti-migratory effect of MNBE was evaluated by using wound healing and Boyden chamber assay. Real-time PCR was used to determine the mRNA expression, and western blot and flow cytometry was used to determine the protein levels of growth and migration-related genes.

**Results:**

MNBE significantly suppressed the growth and survival of cervical cancer cells in a dose-dependent manner by inducing cell cycle arrest and apoptosis. In addition, the growth inhibitory effect of MNBE was specific to cervical cancer cells than normal cells. Cell cycle arrest was correlated to transcriptional downregulation of cyclin dependent kinase 1 (CDK1), cyclin A, and cyclin B. Additionally, MNBE treatment resulted in the upregulation of active caspase-3 protein and downregulation of prosurvival genes, Bcl2, and survivin at mRNA level and NFkB-p65 at the protein level. Furthermore, MNBE inhibited the migration of cervical cancer cells accompanied by modulation of migration-related genes, including zona occludens-1 (ZO-1), matrix metalloproteinase 2 (MMP2), focal adhesion kinase (FAK), N-cadherin, snail, and E-cadherin.

**Conclusion:**

In summary, the present study provides the first evidence of MNBE in restricting cervical cancer cell growth and migration, which warrants further investigation for developing novel anti-cancer drugs.

**Supplementary Information:**

The online version contains supplementary material available at 10.1186/s12906-022-03718-7.

## Introduction

Cervical cancer is the fourth most common cancer worldwide and is considered one of the most lethal gynecological cancer occurring in women. In 2020, nearly 604,127 women were diagnosed with cervical cancer, and 341,831 succumbed to death. In the same year, Asia (including eastern, south-eastern, and south-central Asia) remained at the top of the list to register the highest number of new cervical cancer cases or deaths [1]. Such a large number of cases and deaths in Asia, mainly comprising low- and middle-income countries, are due to population expansion, lack of screening and vaccination facilities, and the high cost of cancer treatment [2]. To date, cytoreductive surgery remains the first line of treatment which is generally followed by chemotherapy [3]. Platinum-based drugs like cisplatin and carboplatin are being used for a long time and have proven effective in cancer treatment. Although at advanced stages, cancer is known to relapse after chemotherapy and acquire chemotherapeutic resistance [4]. Also, conventional chemotherapeutic drugs induce nephro/ototoxicity in patients, thus having dose-limiting side effects [5]. Hence, there is an urgent need to look for other potent and cost-effective antitumor agents with the least or limited side effects.

Medicinal plants hold tremendous potential to cure various diseases and are the reservoir of numerous bioactive compounds with chemopreventive, chemoprotective, and anti-cancer activity [6, 7]. Among the medicinal plants, *Azadirachta indica*, commonly known as neem, is extensively used as traditional medicine in the rural parts of India. The neem tree has gained attention in the scientific community for its insecticidal [8], antimicrobial [9], antiviral [10], immunomodulatory [11], and anti-cancer activity [12]. Various bioactive compounds have been isolated from different parts of the neem tree (leaves, seed, flower, and fruits), including azadirachtin, gedunin, nimbin, nimbolide, and quercetin which have shown strong antineoplastic activity [13, 14]. Studies have also shown the chemo- and radiosensitization effect of neem leaf extract against breast and cervical cancer cells in vitro and in a human neuroblastoma xenograft model, respectively [15, 16]. The chemopreventive potential of neem extract has also been demonstrated in chemical-induced murine carcinoma models [17, 18].

To date, extensive research has been done on elucidating the anti-cancer activity of extracts derived from different parts of the neem tree. To the best of our knowledge, this is the first study that demonstrates the anti-cancer potential of methanolic extract prepared from the stem bark of neem tree against cervical cancer. Although, few studies have reported the immunomodulatory, anti-ulcer, and gastroprotective effects of aqueous neem bark extract [19, 20]. In the current study, we showed that MNBE could restrict the growth of cervical cancer cells by inducing cell cycle arrest, apoptosis and inhibiting cancer cell migration. Though it is unclear which components of MNBE are active in successfully inhibiting the growth of cervical cancer cells, our work with MNBE provides a basis on which further investigation to develop an effective anti-cancer drug against cervical cancer can be executed.

## Material and methods

### Collection of plant material

Stem bark sample of *Azadirachta indica* was collected from the IISER Kolkata campus (22.964638,88.526458), India. *Azadirachta indica*, which grows wildly in India, is not an endangered species; hence no special governmental permission was required to collect the stem bark sample. Authentication of the plant was conducted by Dr. R.K. Gupta, Scientist – ‘E’ and Head of Office at Central National Herbarium, Botanical Survey of India, Howrah, India (voucher specimen no. IISER/JDS-01).

### Preparation of the extract

The MNBE was prepared as described previously [10]. The stem bark sample was washed with double distilled water to remove any dust and foreign materials. Stem bark was shade dried and ground into fine powder. 100 g of neem bark powder was macerated in 150 ml of methanol for one week. On the seventh day, the suspension was mixed vigorously in a shaker at 25 °C for 24 h. The extract was collected by filtering through Whatmann™ filter paper (Grade 1), and the solvent was evaporated using a rotary vacuum evaporator at 55 °C. The dry powder thus obtained was dissolved in Dimethyl sulfoxide (DMSO; cell-culture grade) at a concentration of 100 mg/ml followed by filtration through a 0.22 μm membrane filter and stored in the freezer at − 20 °C until use. DMSO was used as a vehicle in each experiment, and the final DMSO concentration did not exceed 0.1% (v/v).

### Cell culture

Three human cervical cancer cell lines; HeLa, SiHa, and ME-180, and rat normal pulmonary epithelial L2 cells, were purchased from ATCC (American type culture collection). HeLa and SiHa cells were maintained in DMEM and ME-180 cells in RPMI-1640, supplemented with 10% fetal bovine serum (FBS) and 1% penicillin/streptomycin. L2 cells were cultured and maintained in 1X L2 medium (DMEM) supplemented with 10% FBS, 1% 10 mM HEPES buffer solution, 7.5% NaHCO3, 0.1% L-glutamine and 1% penicillin/streptomycin. All cells were maintained at 37 °C in a humidified incubator with 5% CO_2_. All cell culture reagents were purchased from GIBCO.

### Cell viability assay

Cell viability was determined by 3-(4,5-dimethylthiazol-2-yl)-2,5-diphenyltetrazolium bromide (MTT) assay [21]. Briefly, HeLa, SiHa, ME-180, and L2 cells in the logarithmic phase were seeded at a density of 10 × 10^3^ cells/well in 96-well plates (Nunc, Denmark) and incubated overnight. Cells were then treated with different doses of MNBE (0-300ug/ml) for 24 h. Growth medium containing 0.1% DMSO was maintained in parallel as vehicle control in all experiments. After treatment, a complete culture medium containing MTT (Sigma Aldrich) at 0.5 mg/ml was added to each well and incubated for 4 h in a CO_2_ incubator at 37 °C. MTT solution was discarded, and the purple-colored precipitates of formazan were solubilized by adding 100 µl DMSO to each well. Finally, the absorbance was measured at 570 nm in Varioskan LUX (Thermo Scientific, Finland) microplate reader. Each drug concentration was assayed in quadruplicates and repeated three times. The cytotoxic effect of MNBE was expressed as the percentage of viable cells in comparison to vehicle control. Dose–response curve using the log(inhibitor) vs. response—Variable slope (four parameters) was created by GraphPad Prism 5.0 (GraphPad Software, San Diego, CA, USA). The concentration of the MNBE inhibiting cell growth by 25% (IC_25_) and 50% (IC_50_) was obtained and used for further studies.

### Clonogenic assay

To determine the effect of MNBE on colony formation ability of HeLa, SiHa, and ME-180 cells, clonogenic assay was performed. Viable cells were plated in the range of 200–1000 cells depending on the cell type in 60 mm dishes (Nunc, Denmark) and allowed to attach for 24 h. After 24 h, the cells were treated with MNBE at their respective IC_25_ and IC_50_ dose or vehicle (0.1% DMSO) and incubated further for 24 h in a humidified CO_2_ incubator. After 24 h, media was replaced with fresh medium and incubated for another 10–14 days to form colonies. Cells were then fixed with 4% paraformaldehyde and stained with 0.4% crystal violet. Colonies with at least 50 cells were counted manually, and the survival fraction was calculated by dividing the number of colonies that arise after treatment by the number of cells seeded and plating efficiency (PE: number of colonies formed by untreated cells/number of cells seeded) [22]. All treatments were performed in triplicate, and the experiments were repeated three times.

### Cell cycle analysis

To perform cell cycle analysis, HeLa, SiHa, and ME-180 cells were seeded in 6-well plates (3 X 10^5^ cells/well) in their respective culture medium and incubated overnight at 37 °C. The cells were then treated with MNBE at their respective IC_25_ and IC_50_ dose or vehicle (0.1% DMSO) at 37 °C. Following the incubation, cells were trypsinized and washed with ice-cold PBS twice. Cells were then resuspended in 70% ethanol for fixation and incubated for 2 h. Next, cells were washed with ice-cold PBS twice and incubated in PBS containing 50 µg/ml propidium iodide (PI), 100 µg/ml RNase A solution (Thermo Scientific), and 0.05% Triton X-100 for 15 min at 37 °C in the dark, followed by flow cytometric analysis (BD FACSVerse; BD Biosciences) [23]. Cell cycle distribution (G1/G0, S, and G2/M) was determined using FlowJo software (version 10.7.1).

### Apoptosis assay

Apoptotic cells were analyzed using an Annexin V-FITC/PI double staining method. HeLa, SiHa, ME-180, and L2 cells were seeded in 6-well plates (3 X 10^5^ cells/well) in their respective culture medium and incubated overnight at 37 °C. Cervical cancer cell lines were then treated with MNBE at their respective IC_25_ and IC_50_ dose or vehicle (0.1% DMSO) and incubated at 37 °C for 24 h. L2 cells were treated with 50, 100, and 145 ug/ml of MNBE for 24 h. Following the incubation, cells were trypsinized and stained according to the manufacturer’s protocol (eBioscience™ Annexin V-FITC Apoptosis Detection Kit Cat. No. BMS500FI). The stained cells were analyzed within one hour by flow cytometry (BD FACSVerse; BD Biosciences). Data were analyzed in FlowJo software. The percentage of apoptotic cells was quantified by determining the relative amount of Annexin V-FITC positive alone cells (early apoptosis) and both Annexin V-FITC and PI-positive cells (late apoptosis). Both Annexin V-FITC and PI negative cells were considered living cells [24]. The experiments were repeated thrice.

### Wound healing assay

HeLa, SiHa, and ME-180 cells were seeded in 12-well plates in triplicates and allowed to adhere and grow to give a confluent monolayer. At this point, an artificial wound was created with a p200 pipette tip. The reference line was made with an ultrafine tip marker. Cells were then washed gently with PBS and cultured in their corresponding medium with reduced serum (1%) containing non-toxic dose of MNBE i.e., 10, 20 and 30 μg/ml (< IC_20_) or vehicle (0.1% DMSO) for 24 h. Two scratch areas from each well, above and below the reference line, were captured at 0 h, 12 h, and 24 h using an inverted Nikon Eclipse Ts2 microscope (10X) (Tokyo, Japan). Scratch areas were measured using the Image J software. Wound closure (%) was quantified using the percentage change in the normalized measurement area divided by the original open area according to the formula: Wound Closure % = [A (0)—A (t)/A (0)] × 100 where the area (A) at time zero (0) and the area after incubation time (t) were used to calculate the percent wound closure [25].

### Transwell migration assay

The effect of MNBE on cell migration was also assessed by using the Boyden chamber (8 µm pore size, HiMedia, India) [26]. HeLa, SiHa, and ME-180 cells (0.5 X 10^5^ cells) suspended in 250 μl of the serum-free medium in the presence and absence of MNBE were seeded onto the upper compartment of the transwell chamber. The lower chambers were filled with medium containing 10% FBS as an attractant to cause cell migration. The cells were allowed to migrate for approximately 24 h, and non-migrated cells in the top chamber were removed by a cotton swab. Then the migrated cells attached to the lower surface of the transwell membrane were fixed in 100% methanol and stained with 0.4% crystal violet for 15 min. Migrated cells in five randomly selected fields were counted and photographed under a light microscope [21]. The experiment was performed in duplicates and repeated thrice.

### RNA isolation and quantitative real time PCR

To analyze the effect of MNBE on the expression of the cell cycle, apoptosis, migration-related genes qPCR analysis was used. All three cell lines were seeded at a density of 5 × 10^5^ cells/well in a 6-well plate, treated with MNBE at their respective IC_25_ and IC_50_ doses for 24 h. Following treatment, the cells were harvested and washed with 500 μl of PBS. Total RNA was extracted using TRIzol Reagent (Invitrogen, Carlsbad, CA, USA) and quantified by using NanoDrop ND-2000 spectrophotometer. The cDNA was prepared from 1 µg of RNA using a High-Capacity cDNA Reverse Transcription Kit (Applied Biosystems). Quantitative real-time PCR analysis was performed using DyNAmo Color Flash SYBR Green qPCR kit (Thermo Scientific) in CFX96 Touch Deep Well Real-Time PCR System (Bio-Rad) under the following conditions: initial denaturation at 95 °C for 7 min, 40 cycles of denaturation at 95 °C for 10 s, annealing and extension at 60 °C for 45 s. Melt curve analysis confirmed the absence of nonspecific amplification [27]. A sample without cDNA was used as a negative control. Glyceraldehyde-3-phosphate dehydrogenase (GAPDH) was used as an internal control. The crossing point of the amplification curve with the threshold represents the cycle threshold (Cq). The fluorescence threshold Cq values were calculated, and the ΔCq values were determined using the formula ΔCq = Cq target gene – Cq GAPDH. The ΔΔCq values were then calculated based on the formula ΔΔCq = ΔCq treated—ΔCq untreated. The expression level of the target gene in the treated cells was measured relative to the level observed in the untreated cells and was quantified using the formula 2^−ΔΔCq^ [28]. The raw and analyzed real time PCR data is available in additional file I. The primer pairs used are listed in Table I.

**Table I:** List of primer pairs used in real-time PCR

Target genes	Forward primer (5’-3’)	Reverse primer (5’-3’)
GAPDH	CTCCTCCACCTTTGACGCTG	TCCTCTTGTGCTCTTGCTGG
Cyclin A	GCAAACAGTAAACAGCCTGCGTTC	TGGGTCCAGGTAAACTAATGGCTG
Cyclin B	GGAGAGGTTGATGTCGAGCAAC	GAGAAGGAGGAAAGTGCACCATG
CDK1	AGTCTTCAGGATGTGCTTATGCAG	AGAATCCATGTACTGACCAGGAGG
Bcl2	GTGGCCTTCTTTGAGTTCGGTG	GTGCCGGTTCAGGTACTCAGTC
Survivin	GGACCACCGCATCTCTACATTC	ATGGGGTCGTCATCTGGCTC
Snail	TTACCTTCCAGCAGCCCTACGA	GAGCCTTTCCCACTGTCCTCAT
MMP2	ATAACCTGGATGCCGTCGT	AGGCACCCTTGAAGAAGTAGC
FAK	ATCCCACACATCTTGCTGACTT	GCATTCCTTTTCTGTCCTTGTC
E-cadherin	GAAGGAGGCGGAGAAGAGGAC	CGTCGTTACGAGTCACTTCAGG
N-cadherin	AGGTGGAGGAGAAGAAGACCAGG	GATGGCATCAGGCTCCACAGT

### Intracellular flow cytometric analyses

Expression of active caspase-3 and ZO-1 were analyzed using indirect intracellular flow cytometry. Briefly, HeLa, SiHa, and ME-180 cells were seeded in 6-well plates (3 X 10^5^ cells/well) in their respective culture medium and incubated overnight at 37 °C. The cells were then treated with MNBE for 24 h. Following incubation, cells were trypsinized, followed by washing in ice-cold PBS. Cells were then fixed and permeabilized using BD Cytofix/Cytoperm Kit followed by incubation with anti-active-caspase-3 (1:250, BD Biosciences) and anti-ZO-1 (1:200, Invitrogen) antibody. Next, cells were washed twice and incubated in an APC-conjugated (Allophycocyanin) goat anti-rabbit antibody. Cells incubated with only APC-conjugated secondary antibody was used as a negative control. The stained cells were analyzed within one hour by flow cytometry (BD FACSVerse; BD Biosciences). Data were analyzed in FlowJo software. The experiments were performed in duplicates and repeated thrice.

### Protein extraction and western blot

Firstly, cervical cancer cells were treated with MNBE at IC_25_ and IC_50_ doses for 24 h. Then the cells were washed twice with ice-cold PBS and lysed in RIPA buffer containing protease inhibitor cocktail for 30 min on ice. Following centrifugation at 13,300 rpm at 4ºC for 15 min, the supernatant of the lysate was harvested. Protein concentration was determined using the Pierce® BCA protein assay kit (Thermo Scientific, Rockford, IL, USA). Cell lysates containing equal amounts of protein from each sample were subjected to SDS-PAGE and transferred onto polyvinylidene difluoride (PVDF) membranes (Millipore, Bedford, MA). Membranes were blocked for 1 h at room temperature with 5% skimmed milk in TBST (Tris-buffered saline containing 0.1% v/v Tween-20) and then incubated overnight at 4ºC with each primary antibody against p53 (1:1000, BioBharati), p65 (1:1000, BioBharati), GAPDH (1:5000, BioBharati). After three washes in TBST (10 min each), the membrane was incubated with the relevant HRP-conjugated secondary antibodies for 1 h at room temperature, followed by three 10 min washes with TBST. The signal of the bands was detected using SuperSignal West Pico Chemiluminescent Substrate (Thermo Scientific) as per the manufacturer’s instructions. Protein expression was quantified on Image J software. Protein expression levels of target genes were normalized to GAPDH gene expression and expressed as relative fold change compared to vehicle control [21]. Full length uncropped blots are included in additional file II (Fig. S1, S2 and S3).

### Statistical analysis

All experiments were performed at least three times and presented as mean ± SEM. One-way analysis of variance followed by a Dunnett's multiple comparison test were used for comparisons among groups. All statistical analyses were done using GraphPad (La Jolla, CA) Prism 6. A *P* value of < 0.05 was considered statistically significant.

## Results

### MNBE specifically inhibits the proliferation and survival of cervical cancer cells

**Fig. 1:**
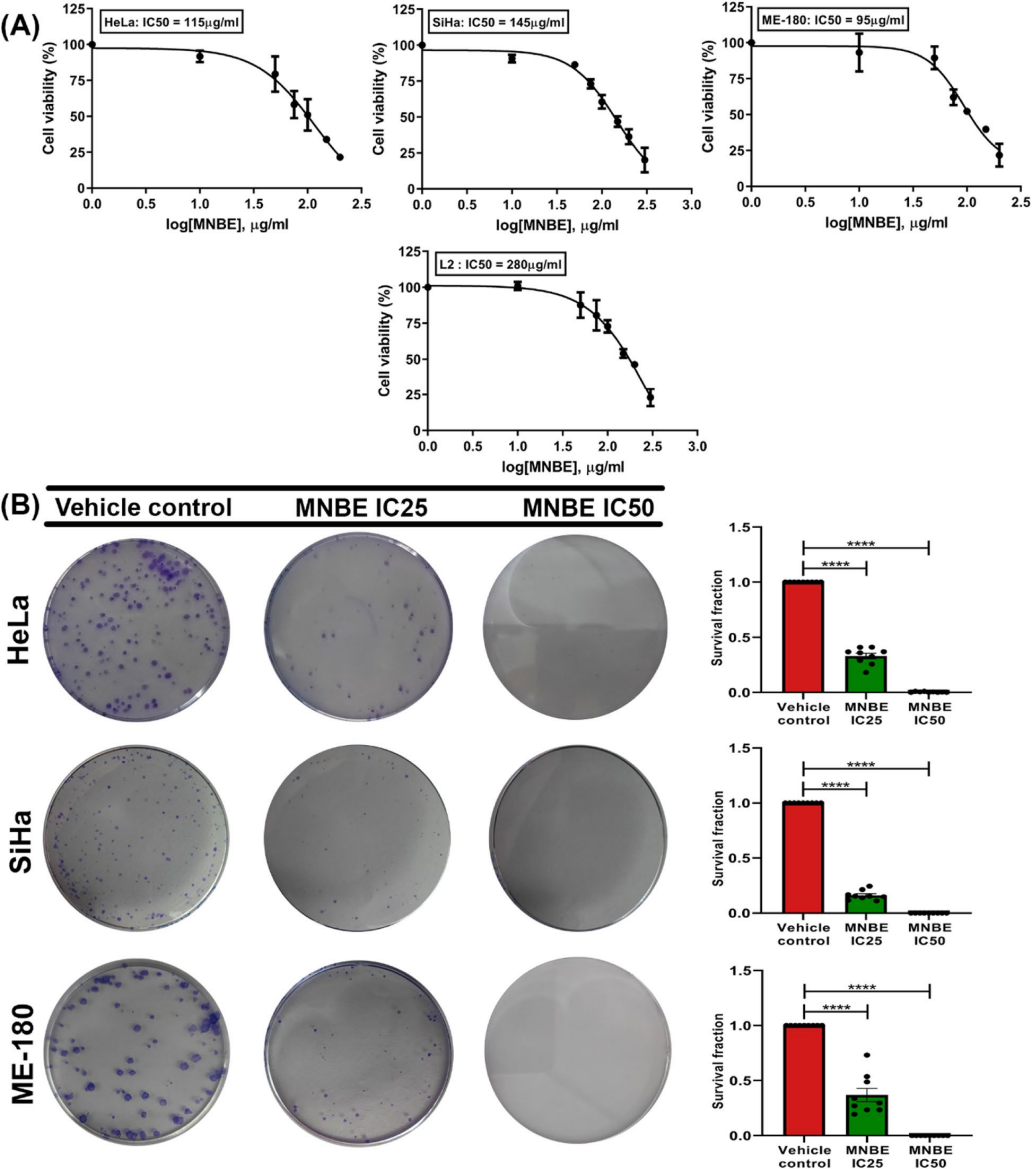
The antiproliferative effect of MNBE on cervical cancer cell lines. (**A**) HeLa, SiHa, ME-180, and non-cancerous L2 cells (10^4^ cells/well) were seeded into a 96-well plate, incubated overnight, and then treated with the 0–300 µg/ml of MNBE for 24 h. Viability was measured by MTT assay, and the viability of untreated cells was set at 100%. Results graphed using the log (inhibitor) vs. response—Variable slope (four parameters) curve using GraphPad Prism. (**B**) HeLa, SiHa, and ME-180 cells were seeded in 60 mm dishes in a range of 200–1000 cells and allowed to grow for 24 h. Cells were then treated with corresponding IC_25_ and IC_50_ doses of MNBE for 24 h. After treatment, media was replaced with a fresh medium, and cells were incubated for 10–14 days. Cells were fixed in 4% paraformaldehyde for 20 min and stained with 0.4% crystal violet for 15 min. The stained cells were viewed and counted under a microscope. All experiments were performed in triplicate, and data are expressed as the mean ± SEM of the three independent experiments. Vehicle-treated cells were used as control. **** *P* < 0.0001 vs vehicle control

To evaluate the effect of MNBE on cell viability MTT assay was performed against a panel of human cervical cancer cell lines, namely HeLa, SiHa, and ME-180. Normal rat pulmonary epithelial L2 cells were used as non-cancerous control. All cells were exposed to various doses (0–300 µg/ml) of MNBE for 24 h. Fig. 1A shows the log dose–response curve of HeLa, SiHa, ME-180, and L2 cells from which the half-maximal (IC_50_) inhibitory concentration of the extract was estimated by nonlinear regression analysis. Results showed that MNBE inhibited the growth of cells in a dose-dependent manner. Interestingly, MNBE had the least inhibitory effect on L2 cells with an IC_50_ dose of 280 µg/ml compared to significantly lower IC_50_ in cervical cancer cell lines; 95 µg/ml for ME-180, 115 µg/ml for HeLa, and 145 µg/ml for SiHa cells. The results indicated that the growth inhibitory effect of MNBE is more specific to cervical cancer cells than normal cells. Further experiments with the cervical cancer cell lines were performed using their respective IC_25_ and IC_50_ doses.

Additionally, clonogenic assay was utilized to investigate the effect of MNBE on the survival of cervical cancer cell lines. HeLa, SiHa, and ME-180 cells were treated with MNBE at their respective IC_25_ and IC_50_ doses for 24 h and allowed to form colonies in a complete growth medium for 10–14 days. As shown in Fig. 1B, relative to vehicle control, cells treated with the extract at IC_25_ dose showed a reduction in cell survival by more than 50%, whereas cells treated with extract at IC_50_ dose showed a reduction in cell survival by more than 95%. Collectively, the results confirmed the anti-cancer potential of MNBE against cervical cancer cells.

### MNBE promotes cell cycle arrest and downregulates the expression of cell cycle-related genes

**Fig. 2:**
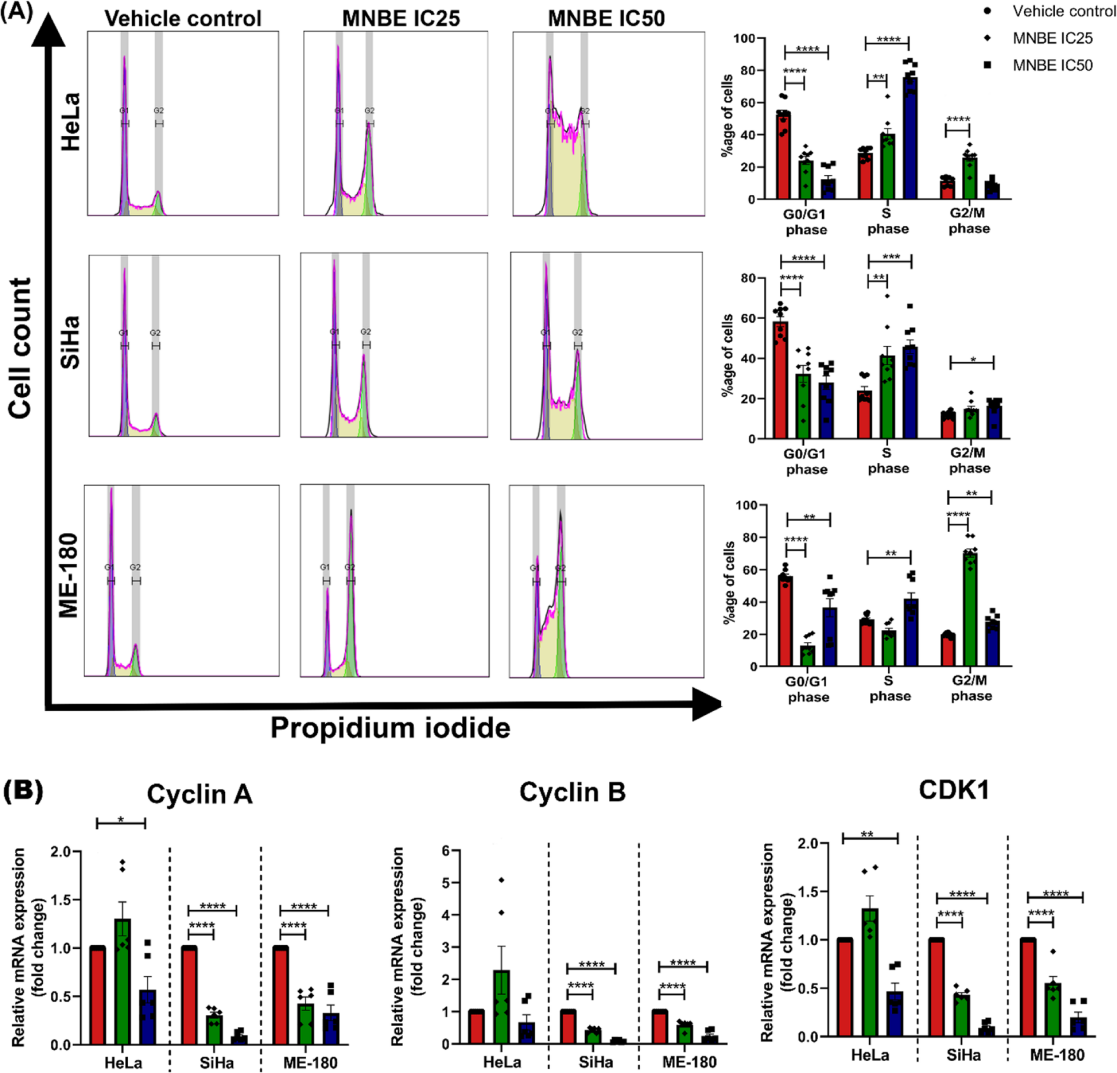
Induction of cell cycle arrest in cervical cancer cells after MNBE treatment. (**A**) HeLa, SiHa, and ME-180 cells were harvested after treatment with MNBE at the indicated concentration for 24 h. The distribution of the cell cycle was analyzed by flow cytometry using PI staining. The percentages of cells in the G1/G0, S, and G2/M phases were calculated using FlowJo software. (**B**) Quantitative real-time PCR analysis of cyclin A, cyclin B, and CDK1 after cells were treated with MNBE for 24 h. Data expressed as the mean ± SEM of the three independent experiments. Vehicle-treated cells were used as control. The significant differences from control are indicated by *P < 0.05, **P < 0.01, ***P < 0.001 and **** *P* < 0.0001 vs vehicle control

To assess whether MNBE exerts its antiproliferative effect by inducing cell cycle arrest, cell cycle distribution was analyzed using flow cytometry. As shown in Fig. 2A, treatment of all three cervical cancer cell lines with MNBE at IC_25_ or IC_50_ dose for 24 h led to a significant decrease in the number of cells in the G1 phase compared to vehicle control. Treatment of HeLa cells with MNBE at IC_25_ dose for 24 h resulted in accumulation of cells in the S-G2/M phase compared to vehicle control. Whereas at IC_50_ dose, a significant proportion of HeLa cells were arrested at the S phase only. Exposure of SiHa cells to MNBE at IC_25_ and IC_50_ doses for 24 h mainly induced S phase arrest. A slight increase in accumulation of SiHa cells in the G2/M phase was also observed at IC_50_ dose compared to vehicle control. MNBE treatment at IC_25_ and IC_50_ dose induced G2/M phase and S-G2/M phase arrest in ME-180 cells, respectively, after 24 h in comparison to vehicle control. Taken together, MNBE treatment resulted in either S, S-G2/M, or G2/M phase arrest depending on the dose of MNBE as well as on the individual cervical cancer cell line.

To investigate the mechanism of MNBE induced cell cycle arrest, we determined the mRNA expression levels of cyclin A, cyclin B, and CDK1 as they are essential for cells to progress through S and G2/M phase. Real-time qPCR analysis showed that MNBE treatment significantly downregulated the mRNA expression levels of cyclin A and CDK1 in all three cell lines, whereas cyclin B was downregulated to significant levels in SiHa and ME-180 cells only (Fig. 2B). Thus, it may be concluded that exposure of cervical cancer cells to MNBE reduced the expression of cell cycle-related genes at the transcriptional level, resulting in cell cycle arrest.

### MNBE promotes apoptosis in cervical cancer cells

**Fig. 3:**
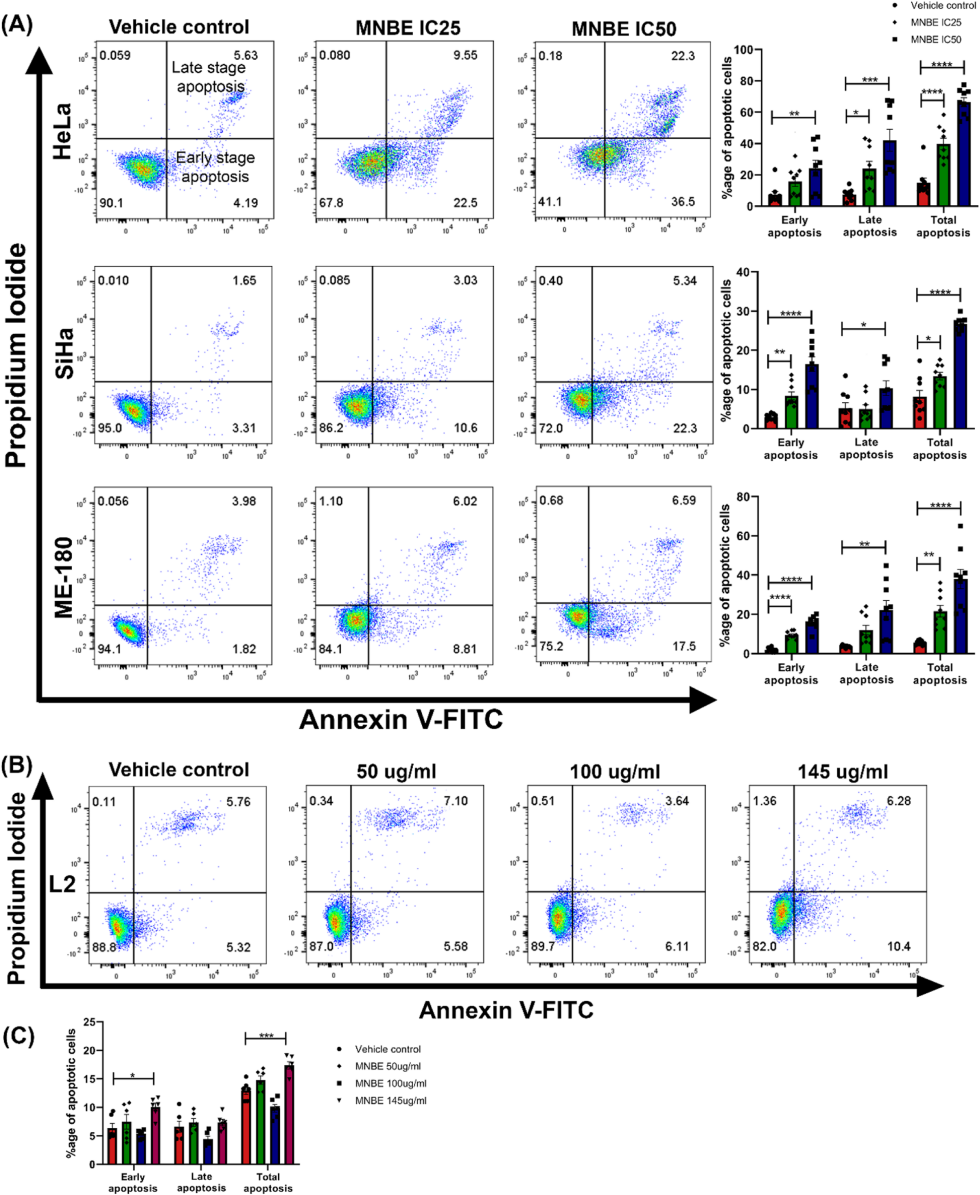
Induction of apoptosis in cervical cancer cells after MNBE treatment. Flow cytometry apoptotic cell analysis of MNBE treated HeLa, SiHa, ME-180, and non-cancerous L2 cells using Annexin V–FITC and PI staining. (**A**) Apoptotic cell death of HeLa, SiHa, and ME-180 cells after treatment with MNBE at their respective IC_25_ and IC_50_ doses for 24 h. (**B**) and (**C**) L2 cells were treated with 50, 100, and 145 µg/ml of MNBE for 24 h. Bar diagrams represent the percentage of apoptotic cells calculated by FlowJo software. Data expressed as the mean ± SEM of the three independent experiments. Vehicle-treated cells were used as control. The significant differences from control are indicated by *P < 0.05, **P < 0.01, ***P < 0.001 and **** *P* < 0.0001 vs vehicle control

To further elucidate whether apoptosis contributes to the growth inhibitory effect of MNBE, HeLa, SiHa, and ME-180 cells were exposed to MNBE at their respective IC_25_ and IC_50_ doses for 24 h. Cells were then stained with Annexin V-FITC/PI double staining and examined by flow cytometry. As shown in Fig. 3A, the results revealed that MNBE dose-dependently induced apoptosis in HeLa, SiHa, and ME-180 cells. In MNBE treated HeLa cells, early apoptosis increased significantly at IC_50_ dose only, whereas a significant increase in late apoptosis was observed at IC_25_ and IC_50_ doses compared to vehicle control. In the case of SiHa and ME-180 cells, MNBE treatment significantly promoted early apoptosis at IC_25_ and IC_50_ doses, whereas late apoptosis increased significantly at IC_50_ dose only. In contrast to the cervical cancer cells, MNBE treatment did not affect the viability of L2 cells at 50 µg/ml and 100 µg/ml concentration although, at a higher dose of 145 µg/ml, there was little induction of apoptosis in L2 cells (Fig. 3B and 3C). These results further indicate the selective cytotoxic effect of MNBE against cervical cancer cells.

### MNBE regulates the expression of proapoptotic and anti-apoptotic genes

**Fig. 4:**
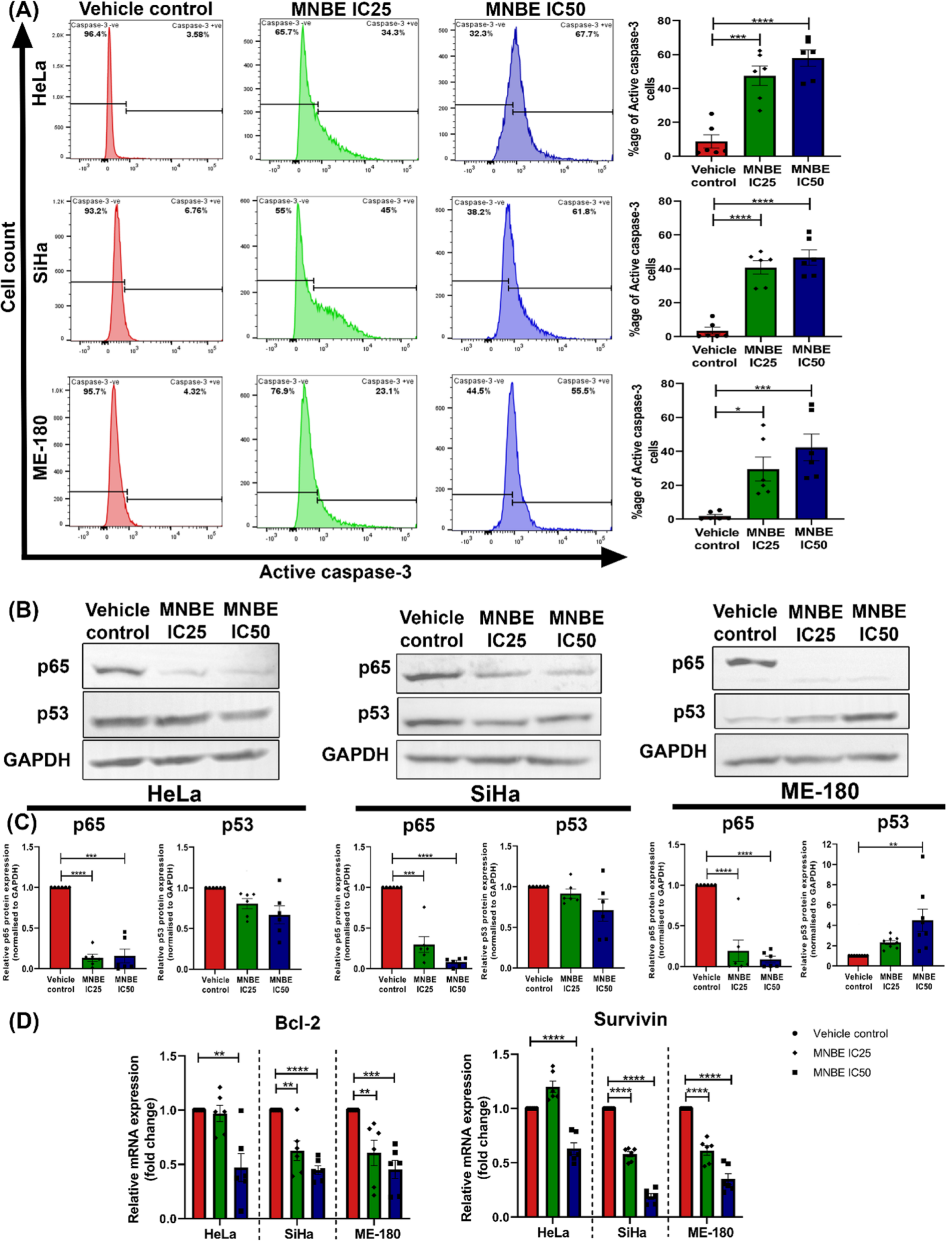
Modulation of pro and anti-apoptotic genes in cervical cancer cells after exposure to MNBE. (**A**) Active caspase-3 expression was detected by flow cytometry in HeLa, SiHa, and ME-180 cells after treatment with IC_25_ and IC_50_ of MNBE for 24 h. (**B**) Western blot analysis of the expression of NFkB-p65 and p53 in HeLa, SiHa, and ME-180 cells treated with MNBE at indicated dose for 24 h. GAPDH in each sample was employed as a standard. (**C**) Quantification of bands of NFkB-p65 and p53 was performed using Image J software normalized to GAPDH. (**D**) Quantitative real-time PCR analysis of Bcl2 and survivin after cells were treated with MNBE for 24 h. Data expressed as the mean ± SEM of the three independent experiments. Vehicle-treated cells were used as control. The significant differences are indicated by *P < 0.05, **P < 0.01, ***P < 0.001 and **** *P* < 0.0001 vs vehicle control

Active caspase-3 is a marker for cells undergoing apoptosis and is responsible for DNA fragmentation and degradation of cellular proteins. To further confirm the apoptotic effect of MNBE, the expression of active caspase-3 was analyzed by flow cytometry. Flow cytometric analysis demonstrated a significant increase in the number of active caspase-3 positive cells in HeLa, SiHa, and ME-180 cells after treatment with MNBE at their respective IC_25_ and IC_50_ doses for 24 h. Moreover, the effect of MNBE on active caspase-3 expression was dose-dependent (Fig. 4A).

We further investigated the effect of MNBE on the levels of p53 protein, an inducer of cell cycle arrest and apoptosis, in HeLa, SiHa, and ME-180 cells. As shown in Fig. 4B, western blot analysis revealed a cell line-specific effect of MNBE on p53 expression. Treatment of HeLa and SiHa cells with MNBE at IC_25_ and IC_50_ doses for 24 h resulted in a slight reduction in p53 levels, though fold change was not considerable. Whereas in ME-180 cells, MNBE treatment resulted in a significant upregulation of p53 levels in a dose-dependent manner with a fold change of 2.3- and 4.5-fold at IC_25_ and IC_50_ dose, respectively.

Since NF-kB is constitutively active in most cancers and contributes to the growth and survival of cancer cells, we examined the expression of NFkB-p65 in HeLa, SiHa, and ME-180 cells after exposure to MNBE for 24 h using the western blot technique (Fig. 4B). Western blot data showed a dose-dependent downregulation in the expression of NFkB-p65 total protein in all the three cell lines after MNBE treatment at IC_25_ and IC_50_ doses (Fig. 4C).

Also, mRNA expression levels of anti-apoptotic genes Bcl-2 and survivin, known targets of transcription factor NFkB-p65, were determined using real-time PCR. As shown in Fig. 4D, treatment with the MNBE significantly downregulated the mRNA levels of Bcl-2 and Survivin in a dose-dependent manner. In HeLa cells, Bcl-2 and survivin were downregulated to significant levels at IC_50_ dose only, whereas, in SiHa and ME-180 cells, there was a significant reduction in mRNA levels of both the genes at IC_25_ as well as IC_50_ dose of MNBE.

### MNBE impedes migration of cervical cancer cells

**Fig. 5:**
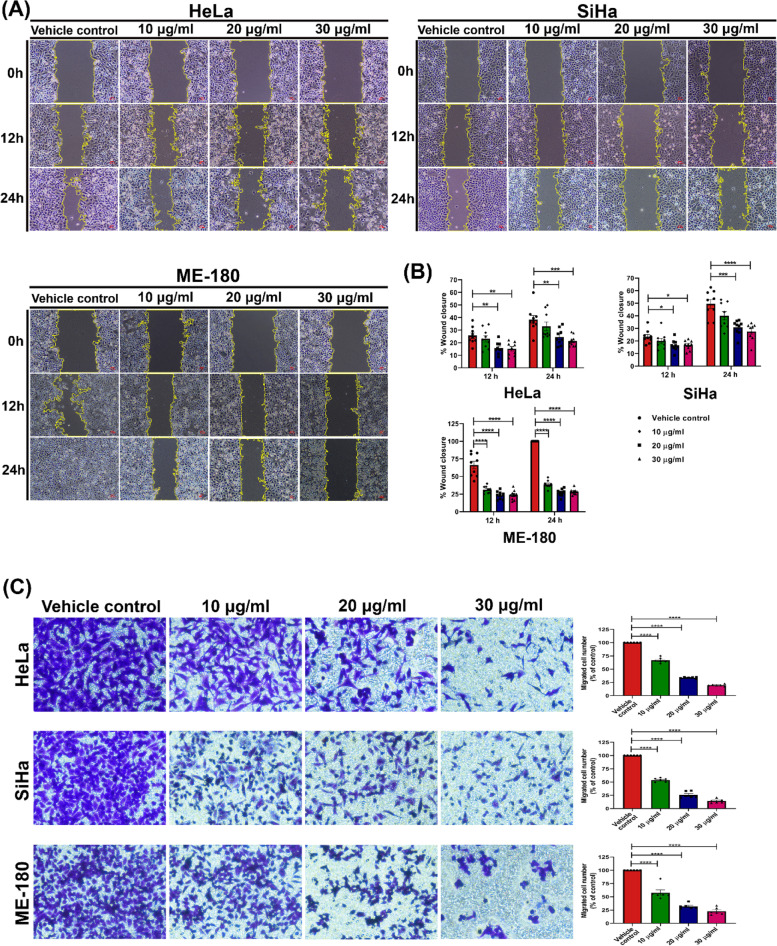
Antimigratory effect of MNBE on cervical cancer cells. (**A**) Microscopical images representing the anti-migratory effect of MNBE on HeLa, SiHa, and ME-180 cells. Cells were incubated in the absence or presence of MNBE at 10, 20, and 30 µg/ml. Images were captured at 0, 12, and 24 h. The yellow lines marked the boundaries of the scratched wounds. (**B**) Results are expressed as the percentage of wound area covered by cells, considering the area measured at 0 h as 0%. (**C**) HeLa, SiHa, and ME-180 cells were seeded in the top chamber of transwell with serum-free medium and treated with 10, 20, and 30 µg/ml of MNBE. After about 24 h, migrated cells were fixed, stained, photographed, and quantified (20X). Data expressed as the mean ± SEM of the three independent experiments. Vehicle-treated cells were used as control. The significant differences are indicated by *P < 0.05, **P < 0.01, ***P < 0.001 and **** *P* < 0.0001 vs vehicle control

Metastasis is one of the hallmarks of cancer and the major cause behind the failure of cancer treatment. As migration of cancer cells is a prerequisite for metastasis, we assessed the effect of MNBE on the migration of cervical cancer cells, HeLa, SiHa, and ME-180, using wound healing and transwell migration assay. As shown in Fig. 5A, in comparison to vehicle control, migration of all the three cell lines into the wound area was significantly suppressed by treatment with MNBE at non-toxic doses, i.e., 10, 20, and 30 µg/ml. Moreover, the effect of MNBE on the migration of cervical cancer cells was dose- and time-dependent as represented in Fig. 5B. Similar results were obtained from transwell migration assay (Fig. 5C), where treatment of HeLa, SiHa, and ME-180 with MNBE for 24 h resulted in a dose-dependent decrease in the number of migrated cells compared to vehicle control.

**Fig. 6:**
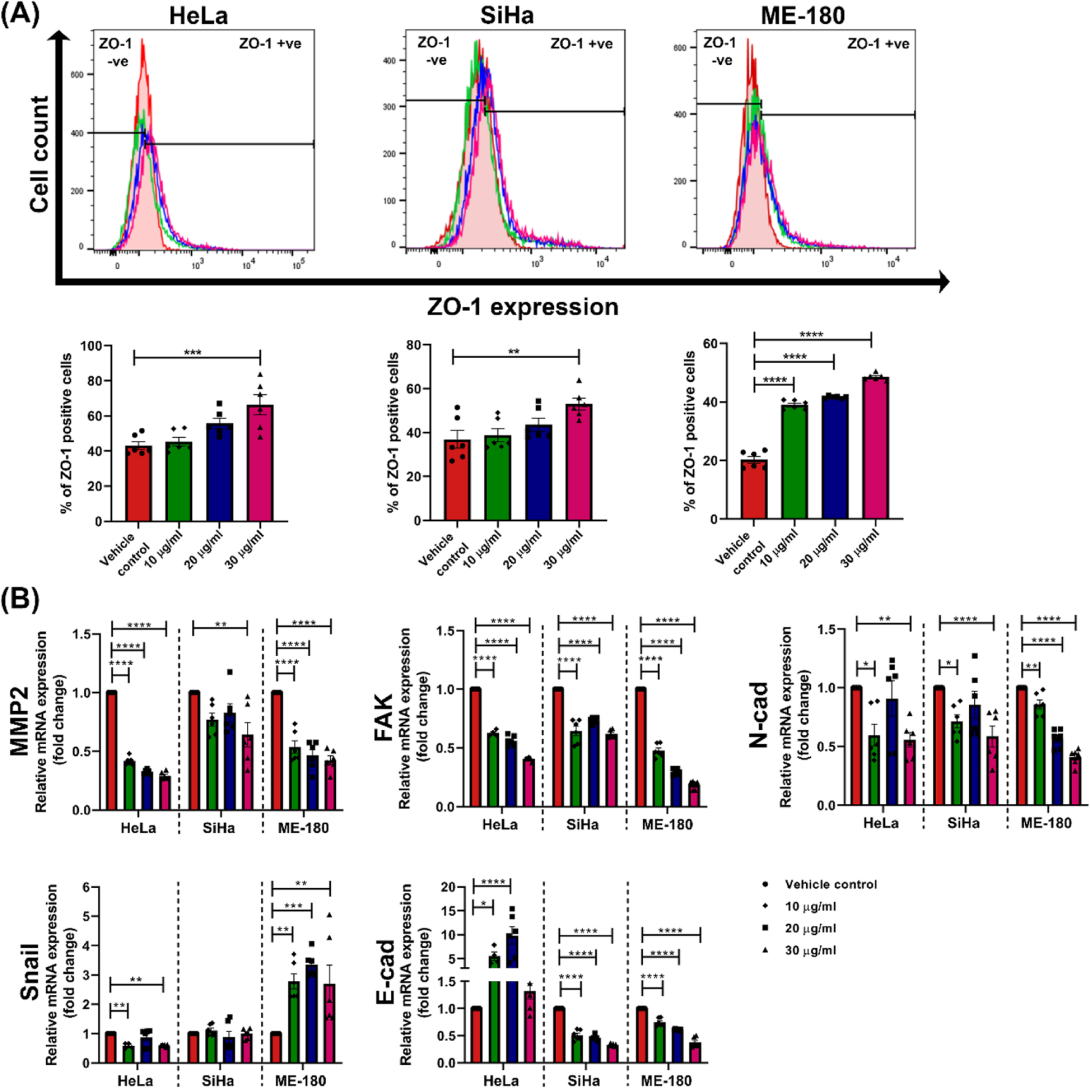
Modulation of EMT markers in cervical cancer cells after MNBE treatment. (**A**) ZO-1 expression was detected by flow cytometry in HeLa, SiHa, and ME-180 cells after treatment with 10, 20, and 30 µg/ml of MNBE for 24 h. (**B**) Quantitative real-time PCR analysis of MMP2, FAK, N-cadherin, snail, and E-cadherin after cells were treated with 10, 20, and 30 µg/ml of MNBE for 24 h. Data expressed as the mean ± SEM of the three independent experiments. Vehicle-treated cells were used as control. The significant differences are indicated by *P < 0.05, **P < 0.01, ***P < 0.001 and **** *P* < 0.0001 vs vehicle control

Given the effect of MNBE on the migration of cervical cancer cells, its underlying mechanism was further investigated. Since cancer cells undergo epithelial-mesenchymal transition (EMT) to acquire migratory and invasive abilities, we sought to determine the expression of EMT-related genes in MNBE treated cells. As shown in Fig. 6A, flow cytometry analysis revealed dose-dependent increase in the number of cells expressing tight junction protein ZO-1 after treatment of HeLa, SiHa, and ME-180 cells with non-toxic doses of MNBE. Moreover, relative transcript levels of other migration-related genes, including MMP2, FAK, and N-cadherin, were significantly downregulated in MNBE treated HeLa, SiHa, and ME-180 cells. Interestingly, there was a cell line-dependent effect of MNBE on the expression of the mesenchymal and epithelial marker, snail and E-cadherin, respectively. The MNBE treated HeLa cells showed significant downregulation in the mRNA levels of the snail with significant upregulation in E-cadherin mRNA levels. On the contrary, MNBE treated SiHa cells showed no change in snail mRNA levels, whereas ME-180 showed a significant upregulation. In addition, MNBE treatment resulted in significant downregulation in E-cadherin mRNA levels in both SiHa and ME-180 cells (Fig. 6B).

## Discussion

In the past few years, numerous studies have shown the chemopreventive and therapeutic effects of neem extracts or their isolated bioactive compounds [12, 14, 18]. Nevertheless, there has been only little scientific advancement in determining the anti-cancer activity of the extract derived from the stem bark of the neem tree. The stem bark of the neem tree is one of the oldest parts and contains various phytochemicals, including di- and tri-terpenoids, C-secomeliacins, polyphenols, and tannins with several pharmacological activities [29]. Hence, in the present study, we explored the anti-cancer potential of MNBE against the panel of human cervical cancer cell lines HeLa, SiHa, and ME-180 and its underlying mechanisms. Our results demonstrated a dose-dependent inhibitory effect of MNBE on the growth and migration of cervical cancer cells. However, the active compounds responsible for the anti-cancer activity of MNBE have not been identified. Hence, it is not clear which active compound/s is responsible for the anti-cancer activity of MNBE or whether it is a combined effect of various phytochemicals present in the extract.

Conventional chemotherapeutic drugs lack the specificity that impacts healthy tissues and limits the efficacy of the treatment. Various plant-derived products have been under extensive investigation and proved to be effective against cancer cells with minimum side effects [6]. In the present study, we showed that MNBE exhibited selective cytotoxicity towards cervical cancer cells compared to non-cancerous L2 cells suggesting the safety of MNBE towards normal cells. Based on the IC_50_ doses of MNBE, the potency of the extract was in the order of ME-180 > HeLa > SiHa > L2 at 24 h time point. Furthermore, clonogenic assay data confirmed the antiproliferative effect of MNBE with a dose-dependent reduction in survival of HeLa, SiHa, and ME-180 cells after 24 h treatment. This led us to further dissect the mechanism behind the inhibitory effect of MNBE on the growth of cervical cancer cells.

Uncontrolled proliferation is the cardinal feature of cancer cells resulting from the dysregulation of cell cycle controls [30]. Many phytochemicals and conventional drugs target the components of cell cycle machinery at critical time points, repressing the proliferation of cancer cells [31, 32]. In fact, all the three cervical cancer cell lines were differentially sensitive to MNBE treatment and showed a dose-dependent effect on cell cycle arrest. Exposure of MNBE to HeLa, SiHa, and ME-180 cells resulted in S, S-G2/M, or G2/M phase arrest depending on the dose of MNBE, which demonstrated a cell cycle phase non-specific nature of MNBE. Few studies have also found similar results using plant extracts [33]. A study by Ozawa et al. showed that the cytotoxic action of cell cycle phase non-specific agents is dependent on concentration and time of exposure [34]. As identification of active compounds in MNBE that accounted for cell cycle arrest has not been made yet, it is difficult to understand whether phase non-specific nature of MNBE is only concentration-dependent or might be due to the action of more than one phytochemical with different targets. Progression through cell cycle phases is regulated by CDKs and their binding partner proteins, cyclins. Dysregulation of CDKs and cyclins has been observed in different types of cancer [35, 36]. CDK1 is known to play an important role in cervical carcinogenesis and mediates the progression of cancer cells through S and G/M phase via binding to cyclin A and cyclin B protein, respectively [36]. In this study, MNBE caused significant downregulation in CDK1 and cyclin A mRNA levels in HeLa, SiHa, and ME-180 cells, whereas cyclin B was only downregulated in SiHa and ME-180 cells. The results indicated that the induction of cell cycle arrest in cervical cancer cells is related to the MNBE mediated downregulation of CDK1, cyclin A, and B.

While performing cell cycle analysis of MNBE treated cervical cancer cells, we also observed accumulation of cells in the sub-G1 phase (data not shown), which is an indicator of apoptosis. To further confirm the results, we stained the HeLa, SiHa, and ME-180 cells with Annexin V-FITC and PI after 24 h treatment with MNBE. Apoptotic cells display phosphatidylserine (PS) on the outer surface of the plasma membrane, and in the presence of calcium ions, Annexin V can bind to PS. Thus, a fluorophore-labeled Annexin V can be used to identify apoptotic cells. To differentiate between the apoptotic and necrotic cells, annexin V is used in conjunction with PI. Early apoptotic cells will bind to Annexin V only, whereas late-stage apoptotic cells and necrotic cells will stain positively for Annexin V and PI due to the entry of PI into the nucleus, where it binds to DNA [37]. In our study, Annexin V-FITC/PI staining revealed the dose-dependent apoptosis-inducing potential of MNBE. The assay further demonstrated the ability of MNBE to induce early and late-stage apoptosis in all three cancer cell lines. Moreover, MNBE did not induce apoptosis in non-cancerous L2 cells at 50 µg/ml and 100 µg/ml concentrations, which were highly toxic to HeLa and ME-180 cells. At a higher concentration of 145 µg/ml, which is the IC_50_ dose for SiHa cells, there was a slight increase in the apoptosis of L2 cells. Together this data suggested an effective and safe therapeutic efficiency of MNBE as an anti-cancer agent.

Caspase-3 is one of the executioner caspases and is an essential mediator of apoptosis. In healthy cells, caspase-3 is present in an inactive proenzyme form which gets proteolytically cleaved and thus gets activated by other caspases in response to apoptotic stimuli [38]. In our study, MNBE dose-dependently increased the levels of active caspase-3 in HeLa, SiHa, and ME-180 cells, further confirming the apoptotic effect of MNBE. The tumor suppressor protein p53 also performs proapoptotic functions and is generally dysfunctional in most human cancers [39]. p53 functions as a transcription factor and exerts its apoptotic effect via inducing the expression of genes known to promote apoptosis and by repressing the transcription of anti-apoptotic genes [40]. It is worth mentioning that the higher sensitivity of ME-180 cells to MNBE treatment compared to HeLa, and SiHa cells might be due to the upregulation of p53 in ME-180 cells which we did not observe in HeLa and SiHa cells. NFkB-p65 is a master regulator required by cancer cells for their continuous proliferation, invasion, survival, and acquiring chemotherapeutic resistance [41]. NFkB signaling pathway is constitutively active in cancer cells and has become a prime target for developing new cancer therapies [42]. Interestingly, we found a dose-dependent reduction in the protein levels of NFkB-p65 in all the three cervical cancer cell lines after MNBE treatment. Furthermore, mRNA expression analysis of prosurvival genes (Bcl2 and survivin), downstream targets of the NFkB-p65 transcription factor, revealed MNBE mediated downregulation in mRNA levels of Bcl2 and survivin in HeLa, SiHa, and ME-180 cells. Bcl2 is an essential regulator of apoptosis and is associated with the prolonged survival of cells by avoiding apoptosis. Bcl-2 prevents apoptosis either by sequestering procaspases or inhibiting the release of cytochrome c and AIF (apoptosis-inducing factor) from mitochondria into the cytoplasm [43]. Survivin, the smallest member of the Inhibitor of apoptosis protein family, can directly bind to active caspase-9 and inhibit the apoptotic pathway in the initial steps [44]. Overexpression of both Bcl2 and Survivin has been reported in different malignancies, including cervical carcinoma [45, 46]. Taken together, the data suggested that MNBE exerts its apoptotic effect via inducing the expression of proapoptotic genes as well as downregulating the expression of anti-apoptotic genes.

Cancer cell migration is one of the critical components of metastasis, the leading cause of death in cancer patients [47]. Therefore, reducing cancer cell migration is one possible approach to prevent cancer metastasis and increase the survival of cancer patients. Cancer cell undergoes EMT to acquire migratory and invasive properties, which involves loss of epithelial markers such as ZO-1, E-cadherin and increased expression of mesenchymal markers such as N-cadherin, vimentin as well as MMPs. During EMT, transcriptional repressors, including Snail, Slug, or Twist, repress the expression of E-cadherin [48]. Furthermore, FAK, a non-receptor protein tyrosine kinase, regulates EMT via intracellular signaling pathways and is overexpressed in different types of tumors [49, 50]. In this study, MNBE severely suppressed the migration of HeLa, SiHa, and ME-180 cells in a time and dose-dependent manner at non-toxic doses. Further mechanistic studies showed that MNBE altered the expression of EMT-related genes, which might be contributing to the inhibition of cancer cell migration. MNBE significantly induced the expression of epithelial marker ZO-1 in all three cervical cancer cell lines. Several reports suggest that the expression of ZO-1 is inversely related to the invasiveness of cancer [51, 52]. Additionally, MNBE downregulated the mRNA levels of MMP2, FAK, and N-cadherin in all three cervical cancer cell lines. In addition, snail, a transcriptional repressor of E-cadherin, was also significantly downregulated in HeLa cells, accompanied by a remarkable increase in the mRNA levels of E-cadherin. However, contrasting results were obtained for snail, and E-cadherin mRNA levels in SiHa and ME-180 cells suggesting the role of other migration-related genes, as significant inhibition in cell migration was observed at the cellular level. Collectively, the data suggested that the inhibitory effect of MNBE on the migration of cervical cancer cells was at least partially associated with the inhibition of EMT via upregulating tight junction protein ZO-1 and downregulating the expression of MMP2, FAK, and N-cadherin gene transcripts.

## Conclusion

In conclusion, this is the first study that demonstrated the anti-cancer potential of MNBE against cervical cancer. MNBE induced cell cycle arrest and apoptosis in cervical cancer cells by modulating the expression of genes involved in cell cycle regulation and apoptosis. Additionally, MNBE impaired cervical cancer cell migration even at non-toxic doses implying the potential of MNBE to treat or prevent cancer metastasis. Nonetheless, further studies are required to identify and isolate the bioactive constituents of this extract that contribute to the anti-cancer activity. Taken together, these findings provide the basis for the further investigation of MNBE against cervical cancer.

## Supplementary Information

**Additional file 1:** .

**Additional file 2:** .

## Data Availability

The datasets used and/or analyzed during the current study are available in supplementary file**.**

## Abbreviations

MNBE: Methanolic neem bark extract
MTT: 3-(4,5-Dimethylthiazol-2-yl)-2,5-diphenyltetrazolium bromide
CDK1: Cyclin dependent kinase 1
ZO-1: Zona occludens-1
MMP2: Matrix metalloproteinase 2
FAK: Focal adhesion kinase
DMSO: Dimethyl sulfoxide
DMEM: Dulbecco's modified eagle medium
RPMI: Roswell park memorial institute
FBS: Fetal bovine serum
PBS: Phosphate buffered saline
PI: Propidium iodide
Annexin V-FITC: Annexin V-fluorescein isothiocyanate
APC: Allophycocyanin
RIPA buffer: Radioimmunoprecipitation assay buffer
SDS-PAGE: Sodium dodecyl sulfate–polyacrylamide gel electrophoresis
TBST: Tris-buffered saline containing 0.1% v/v Tween-20
HRP: Horseradish peroxidase
EMT: Epithelial to mesenchymal transition
