# Supplementary material for: Methanolic neem (Azadirachta indica) stem bark extract induces cell cycle arrest, apoptosis and inhibits the migration of cervical cancer cells in vitro
Source: BMC Complement Med Ther. 2022 Sep 10;22:239. doi: 10.1186/s12906-022-03718-7 (PMC9463741; doi:10.1186/s12906-022-03718-7)
Supplement: Supplementary file 2 — Additional file 2: . [file 12906_2022_3718_MOESM2_ESM.pdf]

# Figure S1

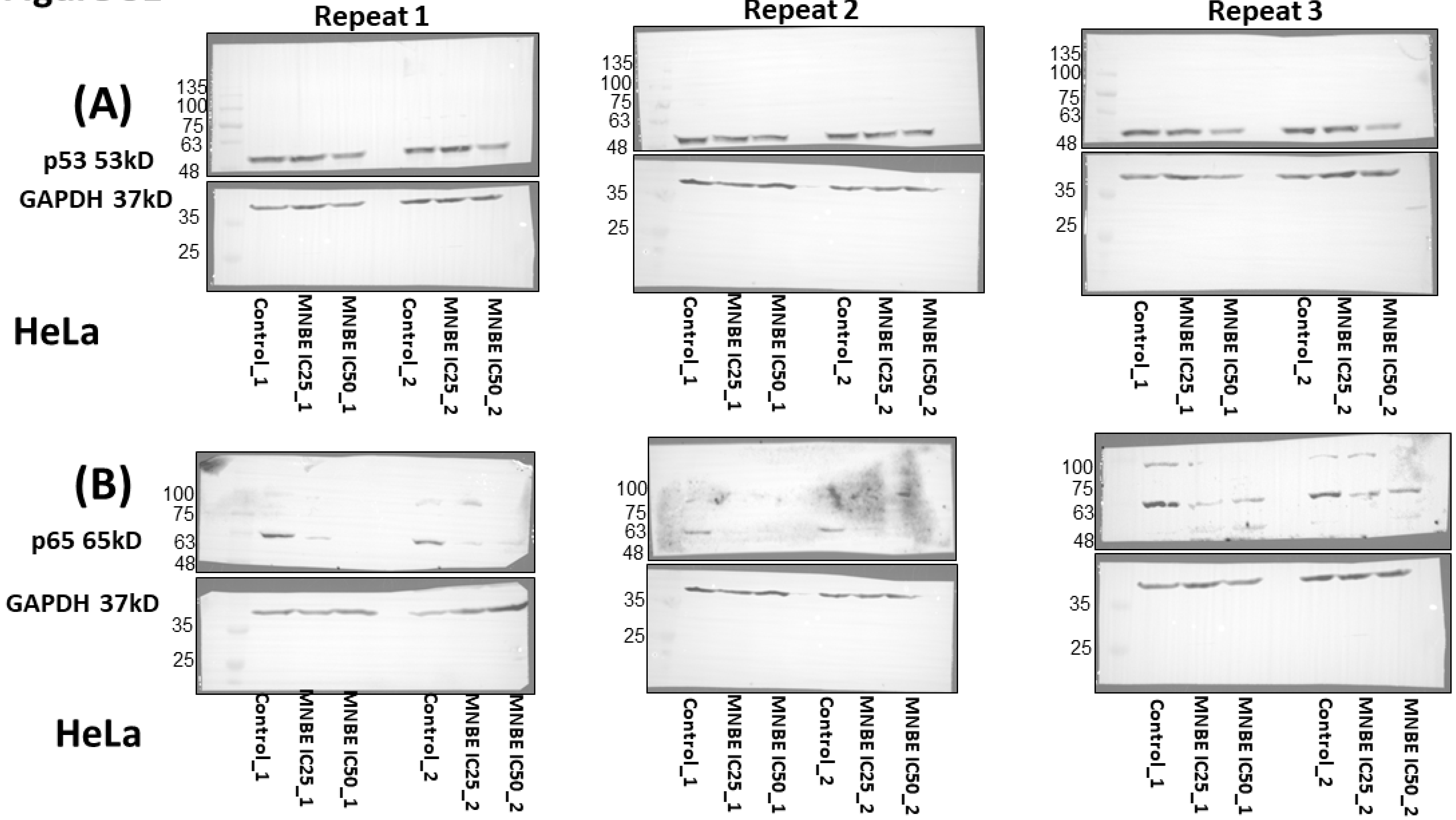


**Figure S1. Original western blots of three repeats for HeLa cells treated with MNBE: (A)** the whole blot after cutting membrane at molecular weight 48kD for P53 (53kD) and GAPDH (37kD). (B) represents the whole blot after cutting membrane at molecular weight 48kD for P65 (65kD) and GAPDH (37kD). (A) and (B) corresponds to the western blot analysis for HeLa cells shown in Figure 4B and 4C of main manuscript.

Figure S2

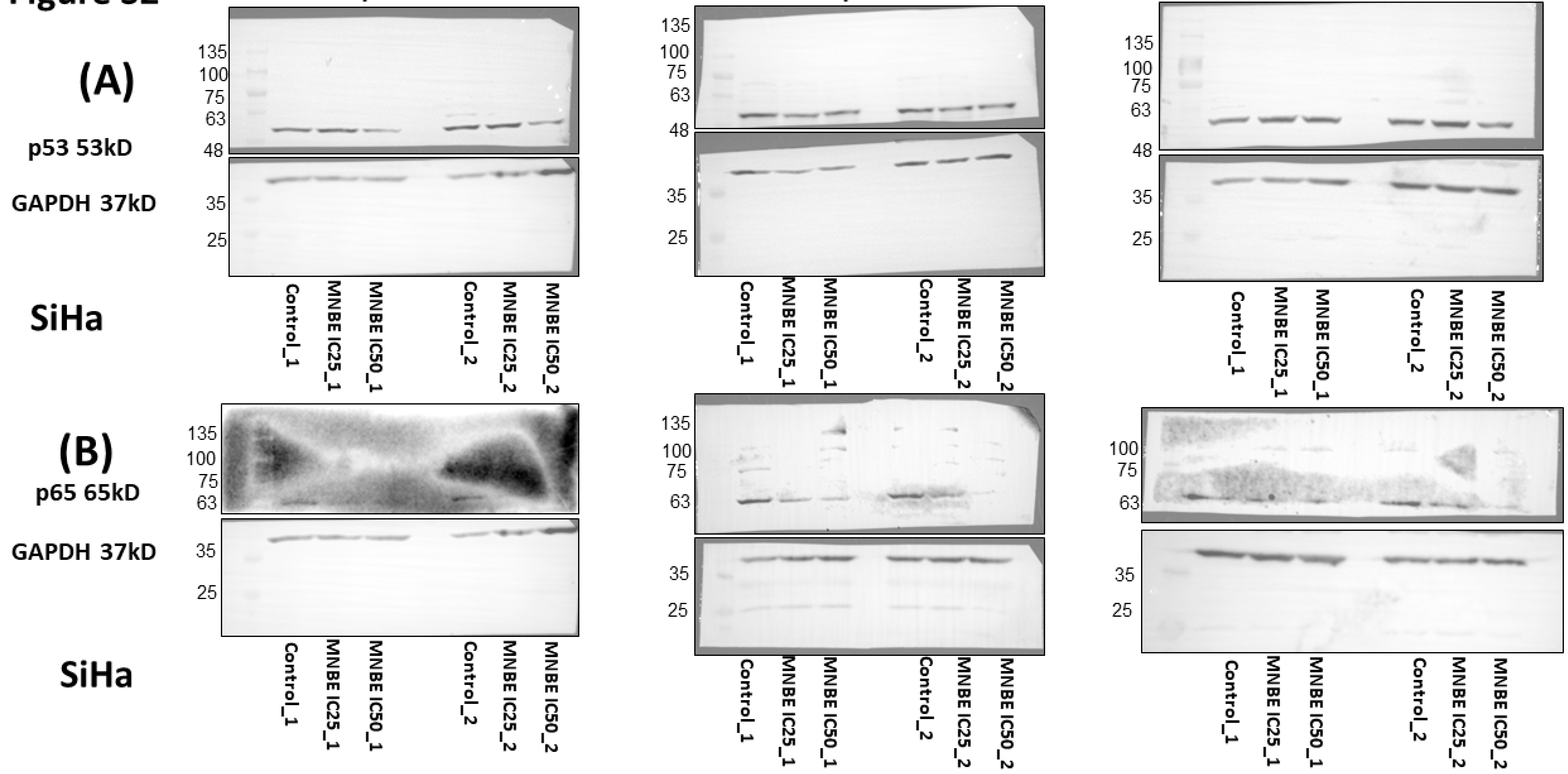


**Figure S2. Original western blots of three repeats for SiHa cells treated with MNBE: (A)** the whole blot after cutting membrane at molecular weight 48kD for P53 (53kD) and GAPDH (37kD). (B) represents the whole blot after cutting membrane at molecular weight 48kD for P65 (65kD) and GAPDH (37kD). (A) and (B) corresponds to the western blot analysis for SiHa cells shown in Figure 4B and 4C of main manuscript.

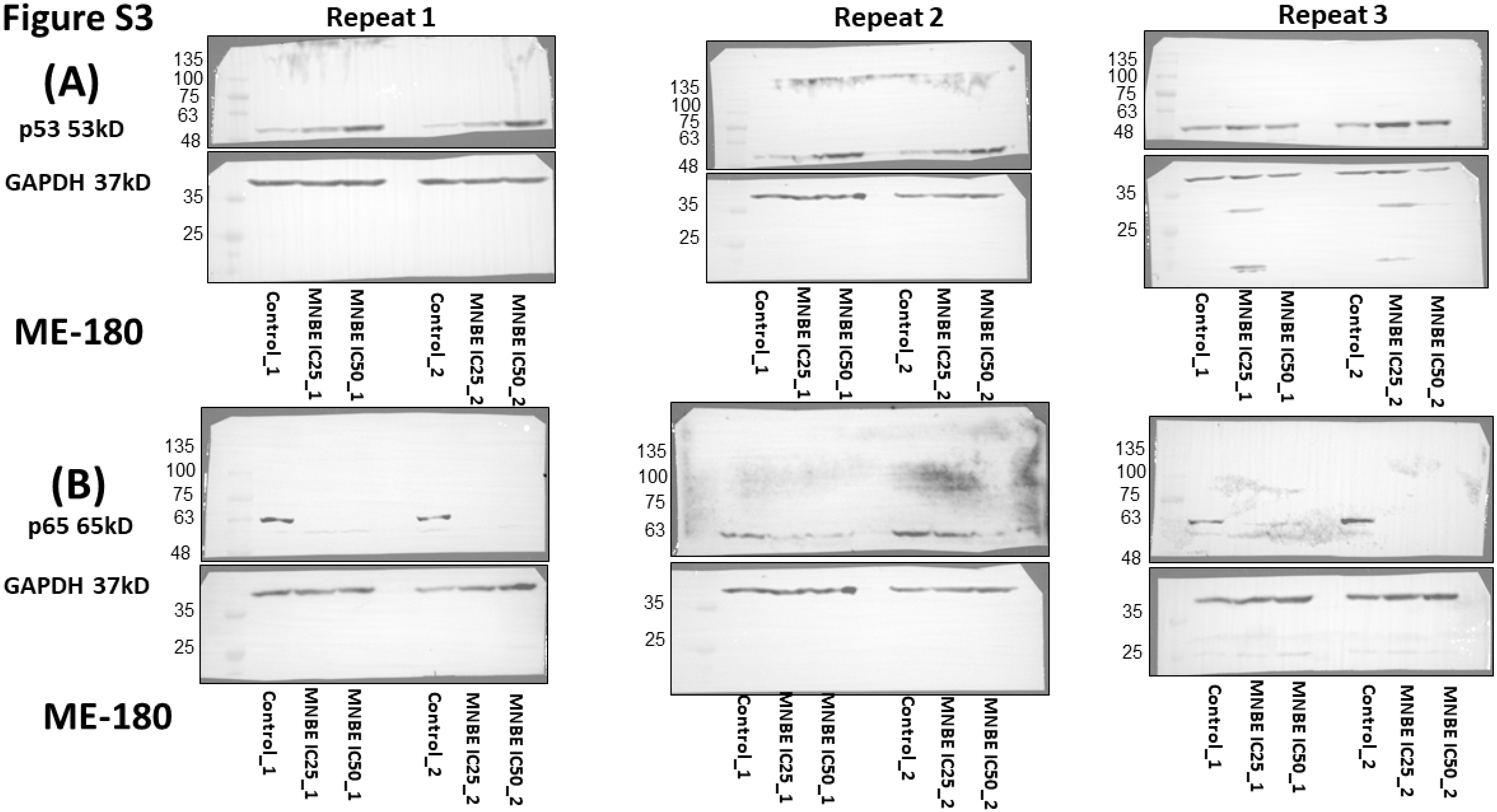


**Figure S3. Original western blots of three repeats for ME-180 cells treated with MNBE: (A)** the whole blot after cutting membrane at molecular weight 48kD for P53 (53kD) and GAPDH (37kD). (B) represents the whole blot after cutting membrane at molecular weight 48kD for P65 (65kD) and GAPDH (37kD). (A) and (B) corresponds to the western blot analysis for ME-180 cells shown in Figure 4B and 4C of main manuscript.
